# Does the use of dynamic elastomeric fabric scoliosis suits provide an improved and more user friendly option for early intervention in childhood scoliosis?

**DOI:** 10.1186/1748-7161-7-S1-P3

**Published:** 2012-01-27

**Authors:** M Matthews, S Bridges

**Affiliations:** 1 DM Orthotics Ltd, Norwich, UK; 2 Ida Darwin Hospital, Cambridge, UK

## Background

The use of dynamic elastomeric fabric orthoses (DEFOs) for the treatment of neurological scoliosis has become an accepted form of orthotic treatment within many leading centres in the UK. The use of rigid or semi- rigid orthoses in this group have proven to be difficult due to reduced acceptance and discomfort. This paper will discuss the use of DEFOs as a first line of orthotic management to obtain improved long term curve management.

## Materials and methods

Children’s skin and rigidity of orthosis construction is often contra- indicated due to low muscle activation and mass. The gold standard use of x-rays, Cobb and off centre alignment informs the orthotic options to couple compression, high pressure and void areas. The DEFO design encourages lateral and de-rotative outcomes to be achieved without rigidity [1-5].

## Results

A child presenting with Myotonic dystrophy, aged 5 years, and 70° right sided thorasic Cobb coupled with a marked pectus carinatum, represents typical results. The Cobb was reduced to 35°, a year later, coupled with improved spinal balance enabling the child to walk unaided for short distance.

Discussion: The use of DEFO in the treatment of early onset scoliosis can have long term corrective outcomes and appears capable of treating higher Cobb angles. The use of DEFOs could also remove the necessity of continual rigid brace management leading to surgery.

## Conclusions

DEFOs could replace rigid and semi-rigid bracing as the preferred treatment modality for children with neurological dysfunction.
